# Imaging characteristics of tenosynovial giant cell tumors on ^18^F-fluorodeoxyglucose positron emission tomography/computed tomography: a retrospective observational study

**DOI:** 10.1186/s12891-023-06730-1

**Published:** 2023-07-19

**Authors:** Kohei Mizuta, Hiromichi Oshiro, Yuichi Tsuha, Yasunori Tome, Kotaro Nishida

**Affiliations:** Department of Orthopedic Surgery, Graduate School of Medicine, University of the Ryukyus, 207 Uehara, Nishihara, 903-0125 Okinawa Japan

**Keywords:** Tenosynovial giant cell tumor, FDG-PET, SUVmax, Intra-articular, Extra-articular

## Abstract

**Background:**

^18^F-fluorodeoxyglucose positron emission tomography/computed tomography (^18^F-FDG-PET/CT) is useful for assessing location, metastasis, staging, and recurrence of malignant tumors. Tenosynovial giant cell tumor (TSGCT) is a benign tumor; however, some studies have reported that TSGCTs have a high uptake of FDG. Few studies have reported on the detailed evaluation of TSGCT using ^18^F-FDG-PET/CT. The purpose of the current study is to evaluate the image characteristics and locations, particularly where possible, with or without, extra-articular invasion from TSGCT of the knee in ^18^F-FDG-PET/CT could occur.

**Methods:**

We retrospectively reviewed the patients with TSGCT who were diagnosed pathologically either by biopsy or surgical specimen. Furthermore, we evaluated the difference of the maximum standardized uptake value (SUVmax) between diffused TSGCT with extra-articular invasion and TSGCT with intra-articular localization in the knee.

**Results:**

The study consisted of 20 patients with TSGCT. The mean SUVmax of TSGCT was 12.0 ± 6.50. There were five patients with TSGCT arising in the knee with extra-articular invasion and six with TSGCT with intra-articular localization. The mean SUVmax of TSGCT with extra-articular invasion and those with intra-articular localization were 14.3 ± 6.00 and 5.94 ± 3.89, respectively. TSGCT with extra-articular invasion had significantly higher SUVmax than TSGCT with intra-articular localization (p < 0.05).

**Conclusions:**

TSGCT revealed high FDG uptake. Furthermore, SUVmax was higher in diffused TSGCT with extra-articular invasion than in intra-articular localized TSGCT; this may reflect its local aggressiveness.

**Supplementary Information:**

The online version contains supplementary material available at 10.1186/s12891-023-06730-1.

## Background

^18^F-fluorodeoxyglucose positron emission tomography/computed tomography (^18^F-FDG-PET/CT) is a tool that combines positron emission tomography (PET) and computed tomography (CT). Generally, malignant tumors have hyper-glycometabolism; hence, PET/CT takes advantage of this property and use it for tumor detection, staging, recurrence, and metastasis [1–3].

It has been reported that ^18^F-FDG-PET is useful for diagnosis, staging, and detecting recurrence of soft tissue tumors [4–10]. Tenosynovial giant cell tumor (TSGCT) is a benign tumor with a high incidence of recurrence. It is classified into diffuse and localized types [11, 12]. Approximately 85% of localized-type TSGCT occurs in the fingers and wrist. However, approximately 75% of diffuse-type TSGCT occurs in the knee joint [13, 14]. The incidence of diffuse-type TSGCT is 4–30%, while the rate of its local recurrence after surgery is 40–60% [13–15]. In patients with diffuse-type TSGCT, joint hemorrhage and repeated local recurrence have been reported to cause joint destruction [11]. On ^18^F-FDG-PET, TSGCT has a high accumulation of fluorodeoxyglucose (FDG) despite it being a benign tumor [16, 17]. However, few reports have been published regarding the detailed evaluation of TSGCT using ^18^F-FDG-PET/CT.

Therefore, the purpose of this study was to evaluate the image characteristics and locations, particularly where possible, with or without, extra-articular invasion from TSGCT of the knee in ^18^F-FDG PET/CT could occur.

## Materials and methods

### Patient selection

This was a retrospective observational study carried out in a single institution between January 2013 and September 2021. The inclusion criteria were patients who underwent ^18^F-FDG PET/CT for diagnosis of bone or soft tissue tumors and patients with TSGCT whose diagnoses were confirmed either by biopsy or surgical specimen. The exclusion criteria were: patients who underwent ^18^F-FDG PET/CT for recurrence or postoperative tumor check-up, patients with incomplete data, and patients with a lack of pathological diagnosis.

### Image acquisition

All PET/CT images were taken with the Biograph mCT (Siemens Healthcare, Japan). After abstaining from food for at least 5 h, patients were intravenously injected with 3.7 MBq/kg of ^18^F-fluorodeoxyglucose (^18^F-FDG). Subsequently, they underwent PET/CT scanning 60 min later. The PET/CT image was included from vertex to toes. The maximum standardized uptake value (SUVmax) was calculated as the highest FDG uptake in the tumor. The SUV is a quantitative index of tissue uptake of ^18^F-FDG and calculated as follows: SUV = PET activity/(injected dose/body weight), where PET activity is a calibrated uptake measured in millicuries per milliliter [18]. Moreover, magnetic resonance imaging (MRI) was performed in all patients with TSGCT in this study.

### Analyses of SUVmax in TSGCT

After image acquisition, values of SUVmax of TSGCT were evaluated. The mean SUVmax of TSGCTs were calculated.

### Comparison of the SUVmax between TSGCTs arising from the knee with extra-articular invasion and those with intra-articular localization

The tumors that invaded the adjacent soft tissues/muscles beyond the joint capsule or bones on MRI were defined as extra-articular invasion. The mean value of SUVmax was analyzed between TSGCTs arising in the knee with extra-articular invasion and those with intra-articular localization as a secondary objective.

### Statistical analyses

Statistical analyses were performed with JMP version 13 (SAS institute inc., Cary, NC, U.S.A.). Data is shown as means ± standard deviations. The student’s t-test was performed to compare the value of SUVmax between TSGCTs with extra-articular evasion and intra-articular localization. Probability (P) values less than 0.05 were defined as statistically significant.

## Results

### Patient characteristics

**Fig. 1:**
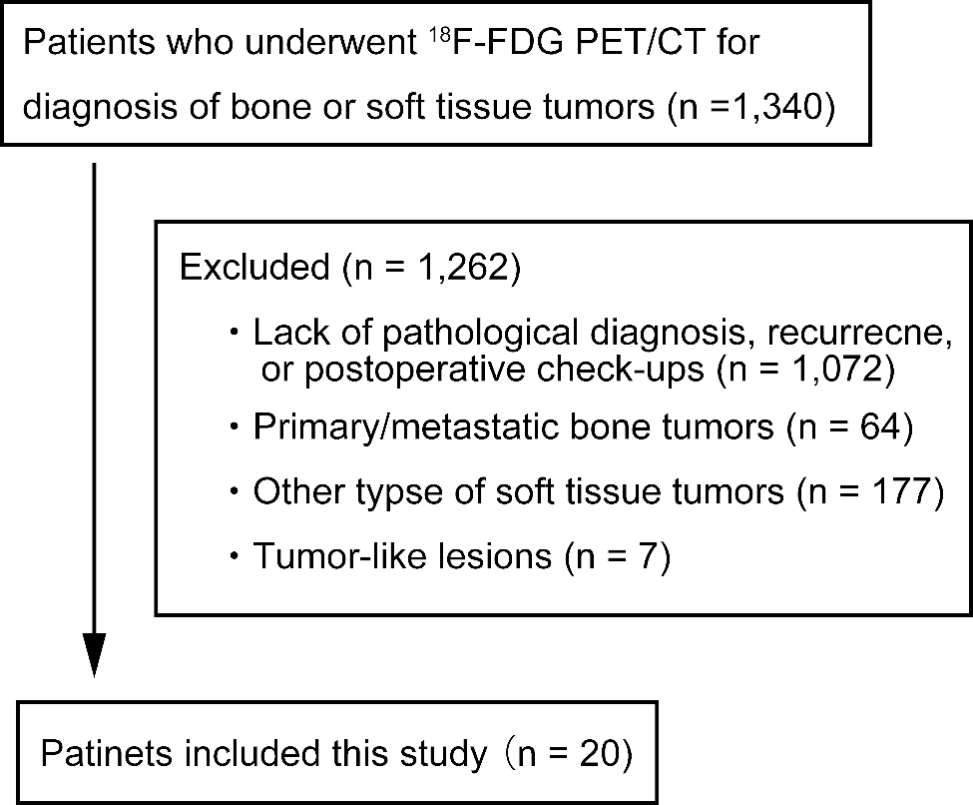
The flow chart of the study design

A total of 1,340 patients who underwent ^18^F-FDG PET/CT for diagnosis of bone or soft tissue tumors between January 2013 and September 2021 in our institution were screened from electronic medical charts. Out of these, 1,262 patients were excluded due to: a lack of pathological diagnosis/recurrence/postoperative check-ups (n = 1,072), primary/metastatic bone tumors (n = 64), other types of soft tissue tumors (n = 177), and tumor-like lesions (n = 7). Hence, 20 patients with TSGCT were included in the study (Fig. 1). The median age was 40.0 years (range, 11–70 years) and the median follow-up period was 16.5 months (range, 0.5–69.1 months). Patient characteristics among TSGCT are shown in Table 1. Thirteen patients had TSGCTs arising from the knee: 11 had the diffused type and two had the localized type. In diffused TSGCT, there were five patients with TSGCT arising in the knee with extra-articular invasion and six with intra-articular localization. Among the patients with TSGCTs, two developed local recurrence. The patient characteristics of those with diffused TSGCT arising from the knee are shown in Table 2.

**Table 1:** Characteristics of patients with TSGCT

Total, n	20
Sex	
Man, n	10
Woman, n	10
Age, median (range)	40.0 (11–70)
Follow-up (mos), median (range)	16.5 (0.5–69.1)

n, number; mos, months; TSGCT, tenosynovial giant cell tumor

**Table 2:** Patient characteristics between diffused TSGCT arising from the knee with extra-articular invasion and those who with intra-articular localization

Total	11
Sex	
Man, n	5
Woman, n	6
Age, median (range)	36.0 (11–70)
Follow-up (mos), median (range)	17.3 (2.3–39.5)
Localization	
Extra-articular invasion, n (%)	5 (45.5)
Intra-articular localization, n (%)	6 (54.5)
Recurrence	
Extra-articular invasion, n (%)	0 (0)
Intra-articular localization, n (%)	1 (9.1)

n, number; mos, months; TSGCT, tenosynovial giant cell tumor

### The SUVmax in TSGCTs

**Fig. 2:**
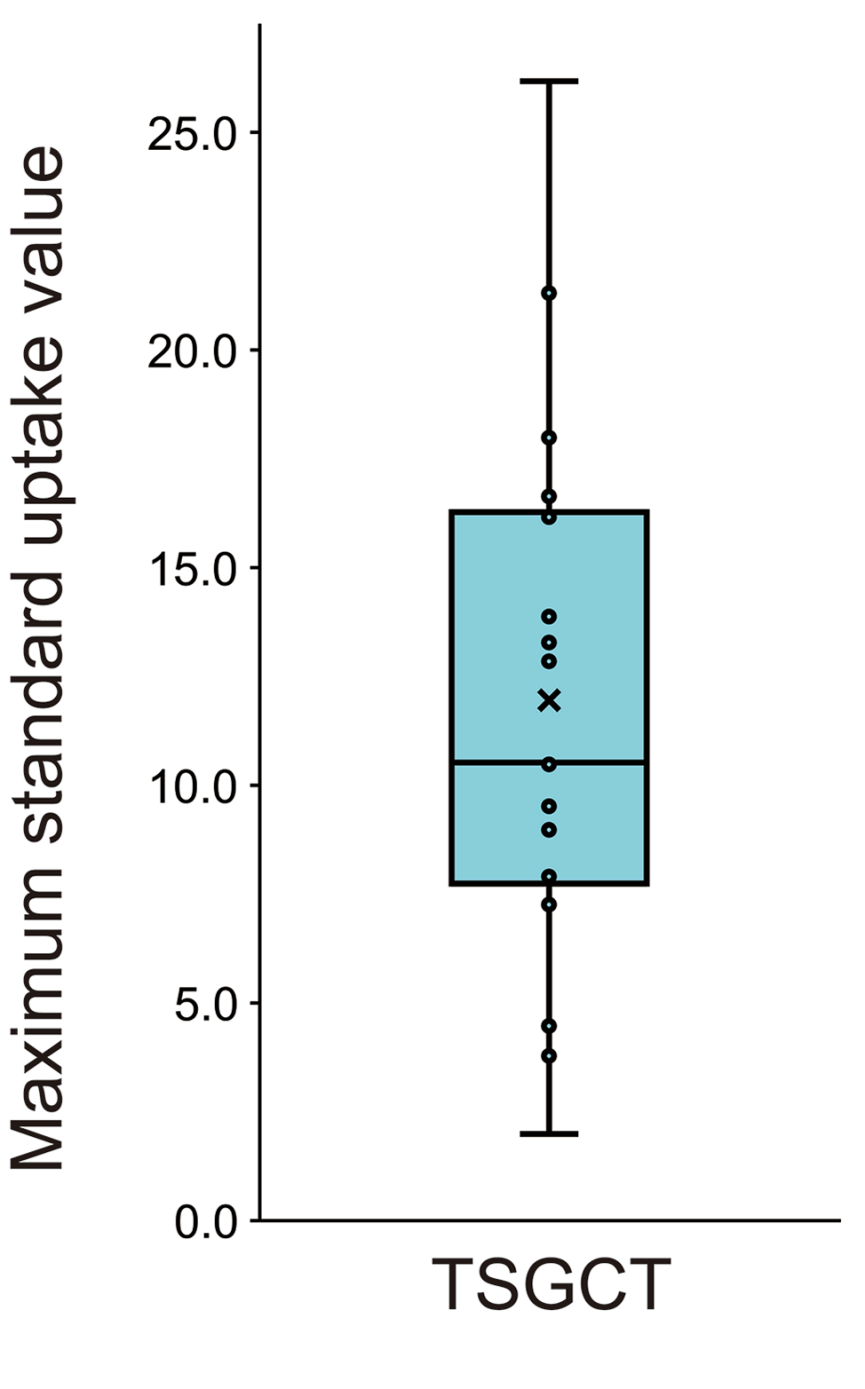
Maximum standard up-take values (SUVmax) in tenosynovial giant cell tumors. Data is shown as box and whisker plots. The center line denotes the median value (50th percentile), while the light blue box contains the 25th to 75th percentiles of dataset. The black whiskers mark the largest and lowest values

The mean SUVmax was 12.0 ± 6.50 for TSGCTs (Fig. 2).

### The difference of SUVmax between TSGCT of diffused type with extra-articular invasion and those with intra-articular localization

**Fig. 3:**
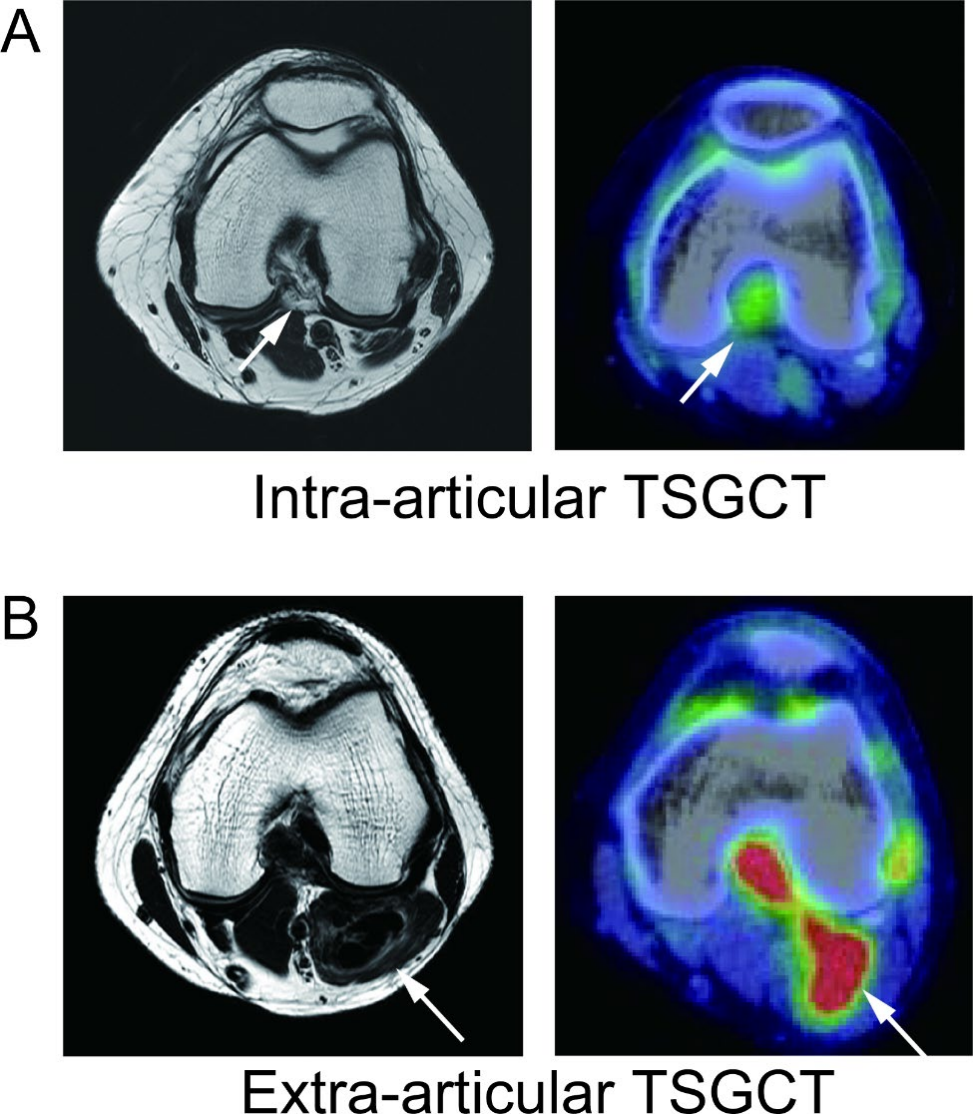
Representative magnetic resonance imaging (MRI) and positron emission tomography/computed tomography (PET/CT) fused images of intra- and extra-articular tenosynovial giant cell tumor. (A) Intra-articular tenosynovial giant cell tumor (*arrow*). *left*, T2 weighted axial view of MRI; *right*, PET/CT fused image (Case No. 5 in dataset). (B) extra-articular tenosynovial giant cell tumor (*arrow*). *left*, T2 weighted axial view of MRI; *right*, PET/CT fused image (Case No. 12 in dataset)

**Fig. 4:**
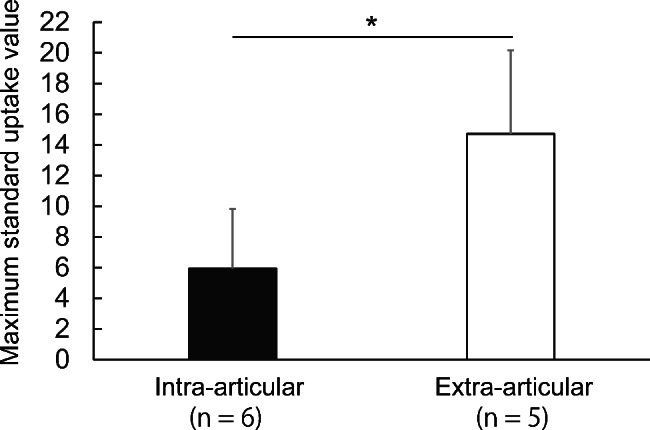
Comparison of maximum standard up-take values (SUVmax) between extra- and intra-articular tenosynovial giant cell tumors. Data is shown as means ± standard deviations. *: p < 0.05 (Student’s t-test)

Five patients had TSGCT arising in the knee with extra-articular invasion and six had only intra-articular localization. Representative MRI and ^18^F-FDG PET/CT scans of extra-articular invasion and intra-articular localization are shown in Fig. 3. The mean of SUVmax of TSGCT with extra-articular invasion and intra-articular localization was 14.3 ± 6.00 and 5.94 ± 3.89, respectively. TSGCT with extra-articular invasion had a significantly higher SUVmax than TSGCT with intra-articular localization (p < 0.05) (Fig. 4).

## Discussion

Our study demonstrated that the SUVmax of TSGCT was significantly high. Furthermore, TSGCT arising from the knee with extra-articular invasion had a significantly higher SUVmax than TSGCT with intra-articular localization.

In spite of being benign soft tissue tumors, TSGCT, elastofibroma, and schwannoma had relatively high SUVmax’s [18]. Some case reports state that TSGCT has a high accumulation of FDG, mimicking malignancy and metastasis [16–21]. The mean SUVmax in patients with TSGCT was higher than the SUVmax in those with benign and non-physiologic pathologies [17]. West et al. reported that TSGCT was related to conditions manifesting features of both reactive inflammatory disorders and clonal neoplastic proliferations involved with abnormal *CSF1* expression [22]. Furthermore, although there are case reports and a small series of TSGCTs, there are no reports that showed a detailed evaluation of TSGCT using ^18^F-FDG-PET/CT focused on the difference of SUVmax between the TSGCT of diffused type with extra-articular invasion and that of intra-articular localization. In this study, the mean SUVmax of TSGCT was 12.0 which was high. In addition, diffused type of TSGCT in the knee with extra-articular invasion had a significantly higher SUVmax than that of TSGCT in the knee with intra-articular localization in this study; this may reflect the local aggressiveness of TSGCT.

This study had several limitations. First, this study was conducted retrospectively in a single institute. Therefore, the sample size was relatively small. Furthermore, subgroup analysis comparing SUVmaxs between intra-articular localization and extra-articular invasion were performed; however, the number of cases in the diffused type of TSGCT in the knee was small. Therefore, future multi-center prospective studies with long-term follow-up and evaluation with a large number of cases are needed.

## Conclusions

TSGCT revealed high FDG uptake. Additionally, diffused type TSGCT of the knee with extra-articular invasion had a higher SUVmax than TSGCT with intra-articular localization which may reflect the local aggressiveness of the disease.

## Electronic supplementary material

Below is the link to the electronic supplementary material.

Supplementary Material 1

## Data Availability

All data generated or analysed during this study are included in this published article and its supplementary information files.

## Abbreviations

^18^F-FDG-PET/CT: ^18^F-fluorodeoxyglucose positron emission tomography/computed tomography
TSGCT: tenosynovial giant cell tumor
SUVmax: maximum standardized uptake value
PET: positron emission tomography
CT: computed tomography
FDG: fluorodeoxyglucose
MRI: magnetic resonance imaging
